# Patient use of blood pressure self-screening facilities in general practice waiting rooms: a qualitative study in the UK

**DOI:** 10.3399/bjgp17X690881

**Published:** 2017-05-09

**Authors:** Alice C Tompson, Sabrina Grant, Sheila M Greenfield, Richard J McManus, Susannah Fleming, Carl J Heneghan, FD Richard Hobbs, Alison M Ward

**Affiliations:** Nuffield Department of Primary Care Health Sciences, University of Oxford, Oxford.; School of Clinical Sciences, University of Bristol, Bristol.; Institute of Applied Health Services Research, University of Birmingham, Birmingham.; Nuffield Department of Primary Care Health Sciences, University of Oxford, Oxford.; Nuffield Department of Primary Care Health Sciences, University of Oxford, Oxford.; Nuffield Department of Primary Care Health Sciences, University of Oxford, Oxford.; Nuffield Department of Primary Care Health Sciences, University of Oxford, Oxford.; Nuffield Department of Primary Care Health Sciences, University of Oxford, Oxford.

**Keywords:** hypertension, primary health care, qualitative research, screening, self-care, self-monitoring

## Abstract

**Background:**

Blood pressure (BP) self-screening, whereby members of the public have access to BP monitoring equipment outside of healthcare consultations, may increase the detection and treatment of hypertension. Currently in the UK such opportunities are largely confined to GP waiting rooms.

**Aim:**

To investigate the reasons why people do or do not use BP self-screening facilities.

**Design and setting:**

A cross-sectional, qualitative study in Oxfordshire, UK.

**Method:**

Semi-structured interviews with members of the general public recruited using posters in GP surgeries and community locations were recorded, transcribed, and coded thematically.

**Results:**

Of the 30 interviewees, 20% were hypertensive and almost half had self-screened. Those with no history of elevated readings had limited concern over their BP: self-screening filled the time waiting for their appointment or was done to help their doctor. Patients with hypertension self-screened to avoid the feelings they associated with ‘white coat syndrome’ and to introduce more control into the measurement process. Barriers to self-screening included a lack of awareness, uncertainty about technique, and worries over measuring BP in a public place. An unanticipated finding was that several interviewees preferred monitoring their BP in the waiting room than at home.

**Conclusion:**

BP self-screening appeared acceptable to service users. Further promotion and education could increase awareness among non-users of the need for BP screening, the existence of self-screening facilities, and its ease of use. Waiting room monitors could provide an alternative for patients with hypertension who are unwilling or unable to monitor at home.

## INTRODUCTION

Blood pressure (BP) self-monitoring is an increasingly common and accepted activity for patients with hypertension, and is usually undertaken in the home.^1^ Meta-analyses of trial data suggest that, after training and as part of an ongoing management plan, self-monitoring leads to clinically significant reductions in BP^2^ and improved medication adherence.^3^ Qualitative studies describe how self-monitoring empowers patients, enabling them to better understand their condition,^4^ although patients need clinician support and education regarding how to interpret results and when to seek assistance.^4^^,^^5^ Some clinicians have also expressed concerns about the potential for bias by patients when reporting self-monitoring data,^6^ and the accuracy of unsupervised measurements^7^ taken on potentially uncalibrated monitors.^8^

BP self-screening whereby members of the public check their own BP using open-access monitors^9^ is now possible with the development of easy-to-use, clinically validated automatic solid-cuff sphygmomanometers.^10^^,^^11^ This type of cuff does not require fitting by a healthcare professional (HCP) and therefore such monitors can be utilised by lay people following simple instructions without training or supervision. BP self-screening offers the opportunity for BP measurements to be made outside their traditional setting of healthcare consultations. It is estimated that BP self-screening kiosks in the US — typically found in pharmacies and work sites — are used over a million times a day.^12^ It has been suggested that the presence of self-screening stations reduces undiagnosed hypertension and improves treatment rates.^9^

A recent systematic review^9^ identified three studies of self-screening: a UK pilot study of BP monitors in community settings;^13^ a Canadian study that extracted the data from BP self-screening kiosks in 341 pharmacies;^14^ and a study of self-screening kiosks in US pharmacies and grocery stores.^15^ Patient experience was investigated in the UK study only. Thirty structured interviews were undertaken with a randomly selected sample of patients who had self-screened a normotensive result. Of these, five (17%) preferred an HCP to measure their BP and 11 (37%) preferred to self-screen, primarily because of the improved convenience, with the remaining participants expressing no preference. Seven (23%) would have liked more privacy. The study did not report whether there were any differences in acceptability depending on the location of the self-screening equipment. Nor did it include people who had not screened.

In a linked study, it was found that the only BP self-screening opportunities routinely available to the general public in an English county were located in GP surgery waiting rooms.^7^ Such facilities are seen by primary care staff and GPs as a method of achieving BP screening performance targets and freeing up clinician time;^7^ however, little is known about how they are viewed by members of the public. The aim of the current study, therefore, was to investigate their reasons for using or not using such facilities to check their BP.

**How this fits in**

Blood pressure (BP) self-screening has been proposed as a way of increasing access to BP checks and reducing clinician workload. This study explores the reasons why patients used or did not use such facilities in GP waiting rooms. The findings will help general practices optimise the use and acceptability of BP monitors in their waiting room.

## METHOD

Semi-structured qualitative interviews were conducted by a non-clinical researcher with members of the general public to gain an in-depth insight into their views and experiences of BP self-screening. Interviewees were recruited via posters displayed in three Oxfordshire GP surgeries with BP self-screening equipment, a library, and a community centre in Oxfordshire. An advert was also placed in an Oxford community newsletter and on their webpage. The posters and adverts invited adults (aged ≥18 years) to contact the research team if they were willing to share their views and experiences, if any, of checking their BP in the community. The researcher then sent them a participant information sheet, consent form, and brief demographic survey for completion and return in a post-paid envelope if they were interested in participating. Recruitment continued until data saturation.^16^ Responders’ demographic details were monitored during recruitment to ensure a balance of ages, sexes, and self-screening experience. Interviewees were offered a £20 gift voucher as an acknowledgement of their time and reimbursed their travel expenses if attending the university for the interview.

An interview schedule was developed based on the study’s objectives and refined after initial use. Topics covered included knowledge and attitudes towards BP, whether they had self-screened, and the process of BP self-screening and other types of BP measurement. Interviews were digitally recorded, transcribed, and checked for accuracy. NVivo (QSR International, version 9) was used to organise the transcripts and record the coding process. A coding framework was developed; initial coding was undertaken independently by two researchers. Codes used were refined using the constant comparison method.^17^ The One Sheet of Paper analysis method was used to compile all the issues raised for a single code and these were grouped to form themes.^18^ The themes that emerged could be grouped into barriers and facilitators.

All interviewees gave written, informed consent before participation.

## RESULTS

The planned sample size of 30 patients was interviewed: 15 (50%) were working, 14 (47%) were female, and six (20%) were hypertensive (Table 1). It was felt that data saturation was achieved. Interviews were conducted on a 1:1 basis apart from a couple who requested to be interviewed together (BPSS111, BPSS112). For logistical reasons, one telephone interview and three interviews were conducted at interviewees’ homes rather than at the university. Box 1 lists the abbreviations used to describe the interviewees.

**Table 1.** Characteristics of interviewees, *n* = 30

	***n***	**%**
BP self-screening service user	14	47

Hypertensive	6	20

Sex: female	14	47

Ethnic group: white	27	90

**Age, years**		
<40	8	27
40–60	12	40
>60	10	33

**Employment status**		
Working	15	50
Retired	7	23
Unable to work	2	7
Unemployed	3	10
Student	2	7
Home/family carer	1	3

Box 1. **Abbreviations used when describing interviewees**

**Table:** 

• M	Male
• F	Female
• Used BPSS	Has self-screened using a waiting room blood pressure monitor
• Not used BPSS	Has not self-screened using a waiting room blood pressure monitor
• HT	Self-reported hypertension
• NT	Self-reported normotension

BP = blood pressure.

Just under half (47%) had used a BP self-screening facility at their own GP surgery. These were typically located in the waiting room or another public area of the building, such as a vestibule. Service users had mostly found the equipment simple to use and described self-screening to be a positive experience in the interview.

Below the interviewees’ reasons for and against checking their BP are described.

### ‘Cos it was there’

The main stimulus reported for using the equipment was observing it while waiting for their appointment with an HCP. There were few alternative activities with which to fill their time:

*‘It’s either go and sit and read a magazine … or go and do something that might be beneficial for your health.’* (BPSS112, female [F], 25 years, used blood pressure self-screening [BPSS], normotensive [NT, self-reported])

Interviewees with no history of elevated readings had little concern about their BP. They self-screened to confirm their continued healthy status or used it as something to fill the time spent waiting:

‘It’s entertaining.’

(BPSS109, F, 50 years, used BPSS, NT)

### Helping the doctor

There was a general feeling that checking their BP was a good thing and that the results were of use to HCPs, if not themselves. Asked why he had used the self-screening monitor while waiting for his appointment, one interviewee explained it was to assist his GP in making a diagnosis, that is:

‘To give the doctor as much information about me as possible.’

(BPSS101, male [M], 57 years, used BPSS, NT)

BP self-screening was felt by patients to free up the time that would have otherwise been spent conducting the measurement in a time-restricted encounter:

‘*If you’ve gone with a list of other things to be dealt with and it’s yet one more thing to add to the GP’s list, yet it’s a thing you can do yourself.’*

(BPSS110, F, 52 years, used BPSS, NT)

Requesting a BP measurement from an HCP may trigger questioning, and if the result was normotensive this was interpreted as wasting the HCP’s time. Some patients, whose BP was being monitored because of potential side effects of their medication, for example, were asked by their GPs to use the equipment before their next appointment. Another interviewee’s GP noticed:

‘Oh we haven’t really checked everything over for lots of years, can you go and go on the weighing machine and the blood pressure machine and hand the tickets back into the receptionist?’

(BPSS107, F, 45 years, used BPSS, NT [patient here reporting the remarks of their GP])

The interviewee had previously used the BP machine out of curiosity but had not disclosed the result to her GP as it was not elevated.

### Taking control

One key perceived benefit was the avoidance of the perceived white-coat effect (WCE),^19^ especially among those with a self-reported history of elevated results:

‘Whoosh! It goes up because stupidly you get nervous, it’s silly.’

(BPSS104, F, 67 years, used BPSS, hypertensive [HT, self-reported])

During a consultation, the HCP controls the initiation of the BP measurement, interpreting and responding to the result:

‘When they say “Ah we’ll just do that again, just to make sure”, you think, “No it’s just because you can’t believe me reading.”’

(BPSS105, M, 58 years, used BPSS, HT)

In addition to being anxious about the result, some disliked the sensation of the cuff tightening:

*‘My partner has a bit of white-coat syndrome and* [by self-screening] *he was trying to get into being comfortable using it and monitoring and just trying to de-sensitise himself.’*

(BPSS109, F, 50 years, used BPSS, NT)

Service users felt empowered by being able to decide when and how often to check their BP:

‘I can just sort of walk in and do it without asking and that really helps.’

(BPSS122, F, 46 years, used BPSS, NT)

Patients with hypertension, in particular, were able to manage what they disclosed to their HCP and what was recorded:

*‘You can give it* [the result] *to the receptionist and they’ll enter it into your record if you want to or screw it up and not tell them if you think you’re stressed out.’*

(BPSS122, F, 46 years, used BPSS, HT)

For those on the cusp of being diagnosed with hypertension, BP self-screening provided the opportunity to challenge the impending diagnosis:

‘*Just give me a chance, I don’t want to take any medication yet because I just feel that I don’t have any blood pressure problem.’*

(BPSS129, F, 35 years, BPSS user, NT)

### A superior measurement over home monitoring BP?

An unanticipated finding was that several interviewees had discontinued home BP monitoring, preferring instead to use the waiting room machine. This was partly because of concerns about accuracy of home readings:

‘I prefer really to use the surgery one because I think in my head that that’s going to be more accurate.’

(BPSS104, F, 67 years, used BPSS, HT)

Fluctuations in home results were interpreted as being caused by deficiencies in their equipment or measurement technique:

*‘I’m sure that it* [the waiting room monitor] *must be better than this sort of little piece of kit I bought on eBay for £10 or whatever I paid for it, you know, it looks a little bit more professional.’*

(BPSS105, M, 58 years, used BPSS, HT)

Others did not want to introduce the negative feelings associated with BP measurement into their home or to facilitate excessive measurement:

*‘The GP suggested that he* [the interviewee’s partner] *get a blood pressure machine of some sort for home use and we talked about that and weren’t quite sure that that was a good idea just because it could become even worse, just, you know, constantly checking and not having it* [his BP] *do what he wanted.’*

(BPSS109, F, 50 years, used BPSS, NT)

In general, interviewees felt reassured by the robust monitor that they were not responsible for maintaining:

‘*They wouldn’t have an inaccurate machine in a doctor’s surgery, I mean that’d be criminal really.’*

(BPSS101, M, 57 years, used BPSS, NT)

The solid cuff in the surgery that did not require adjustment was also preferred:

‘*It was easier to stick it in than to put a cuff on.’*

(BPSS124, F, 84 years, used BPSS, HT)

### Limited awareness of facilities

A key issue in using the equipment was knowing it was available. Promotion by GP practices varied:

‘It wasn’t pointed out that this machine was available, you just had to find out yourself.’

(BPSS101, M, 57 years, used BPSS, NT)

Infrequent attenders were unaware that the service was available, with several interviewees voicing reluctance to visit the GP:

*‘I don’t tend to go to a GP ‘cos obviously I’m male and British so I’ll die first rather than go* [laughs].’

(BPSS125, M, 50 years, not used BPSS, NT)

### No need to check

Linked to the limited understanding of the asymptomatic nature of hypertension, some felt they would sense if something was amiss and therefore BP screening was not required:

‘I think I would know if it gets too high … I think if there was something wrong with my blood pressure I would be aware.’

(BPSS113, M, 67 years, not used BPSS, NT)

Regarding a friend who had been diagnosed with hypertension, one interviewee described how:

‘... *there’s nothing wrong with the woman … she looks fit, healthy … she’s great … how can she be* [hypertensive]*?’*

(BPSS127, F, 79 years, used BPSS, NT)

Some were reluctant to use the self-screening equipment because BP was not on their ‘agenda’ for attending the surgery:

*‘She* [interviewee’s mother] *just thought it was pointless … I guess she just felt, feels healthy and … she wasn’t going to the doctor with concerns about her blood pressure.’*

(BPSS112, F, 25 years, used BPSS, NT)

Female interviewees also described having their BP checked as part of their routine health care, reducing the need to check it themselves:

‘When you’re pregnant and that sort of thing you get your blood pressure taken a lot.’

(BPSS107, F, 45 years, used BPSS, NT)

### The job of a clinician

Although its novelty attracted some to use the monitor, its differing design from monitors used by HCPs or at home deterred others:

‘*I didn’t know how the cuff was going to inflate or whether it was going to be painful or not.’*

(BPSS111, M, 51 years, used BPSS, NT)

Others were concerned that a monitor designed for public use had been oversimplified and would produce poorer results than those suitable for expert use:

‘Our only association with blood pressure is a qualified health professional, often a doctor, doing it for you so you think if the doctor can only do that, I can’t.’

(BPSS125, M, 50 years, not used BPSS, NT)

Some doubted their ability to achieve a reliable BP result or interpret it correctly. One interviewee described how having her BP measured by a clinician was in itself healing:

‘There’s something quite kind of therapeutic about the healthcare professional putting on a blood pressure cuff, blowing it up, reading out the numbers, it just seems like kind of, I can’t really explain it … like the personal touch is really important.’

(BPSS116, F, 30 years, not used BPSS, NT)

### Being on show

Being observed and having to remove outdoor clothing meant a minority of interviewees felt unable to use the self-screening equipment:

‘You have to get up and walk over the waiting room and perhaps take some of your clothes off, particularly if it’s the winter.’

(BPSS107, F, 45 years, used BPSS, NT)

A younger interviewee was concerned it might highlight to others that she had an ongoing (unseen) health condition:

‘*I think people might have thought I was just messing around if I’d come in to do it myself in the waiting room.’*

(BPSS116, F, 30 years, not used BPSS, NT)

## DISCUSSION

### Summary

For those with no history of hypertension, the decision to check their BP was made lightly with little consideration of their susceptibility or any potential consequences. They self-screened to fill the time waiting for their appointment and to confirm their normotensive status. For patients with hypertension or those with a history of elevated results, the process was more considered. By self-screening, they gained control by deciding when they checked their BP and whether to disclose the results to their HCP.

Although BP self-screening removed the presence of an HCP, it introduced new anxieties associated with measuring BP in a public place, such as doubts about ability to use the equipment and about interpreting the results, which formed barriers to self-screening.

### Strengths and limitations

To the authors’ knowledge this is the first qualitative study investigating patient reasons for using public BP self-screening facilities. A recent systematic review of hypertension detection identified few existing studies that addressed barriers to attending screening, possibly because of the difficulty of conducting population-based research rather than using the ‘captive’ populations within hypertension clinics.^20^

Although the predominantly white sample reflects the ethnic make-up of Oxfordshire,^21^ it limits the generalisability of the study findings to more diverse settings. Public BP measurement — including the removal of outer clothing — may be less acceptable in other cultural groups: the BP-Eth study, conducted in Birmingham (UK), found that people of South Asian and African Caribbean ethnic groups rated BP monitoring to be less acceptable than white English people.^22^ This included ambulatory, office, and self-monitoring measurements, although the acceptability of measurements taken in the waiting room was not studied.

The present sample included six self-reported patients with hypertension and several more who described themselves as borderline. No interviewee had their first experience of an elevated reading using the waiting room monitor, however, and therefore future studies could explore the experience of self-screening triggering a change in BP status and the impact of not having an HCP present at this time.

### Comparison with existing literature

BP self-screening users accepted measurements taken in the waiting room as accurate, with some believing them to be superior to home readings, or free from the WCE. Although the diagnostic and prognostic accuracy of home BP measurements^23^^–^25 and the phenomenon of the WCE^26^ are well characterised, the present authors are aware of only one study examining the accuracy of BP measured in a waiting room.^27^ That study found such measures were comparable with the ‘gold standard’ of ambulatory BP measurements, but the results are of limited comparability with those of the current work as the previous study used a BpTRU device not a solid-cuff monitor that had been specifically designed for waiting room use. Another trial showed that regular use of self-monitoring in the waiting room led to lower BP, at least in the short term.^28^

Those who rarely attended their surgery or were less engaged with their health were unaware of the availability of open-access BP monitors in their GP surgery. Several male interviewees expressed reluctance to attend their surgery. GP consultation rates are known to be higher in females than males, significantly so in younger adulthood (15–44 years).^29^ Meanwhile, national surveys have shown that, in those aged <70 years, hypertension is more prevalent in males than females,^30^ suggesting a mismatch between the need for screening and accessibility of waiting room BP monitors, which may have little promotion outside the GP practice.^7^

Some interviewees felt that self-screening was unnecessary as they would sense if their BP was elevated. The perceived symptomatic nature of hypertension has been reported elsewhere, as has the close link between elevated BP and stress.^31^ Among clinicians, BP is an accepted measure of cardiovascular health, but patients may be using self-screening to monitor other parameters of their wellbeing such as their stress levels. The present findings also concur with a systematic review that concluded a lack of knowledge regarding the importance of BP screening and the potential consequences of hypertension were the most common barriers to hypertension detection.^20^

The present finding that some patients preferred to monitor their BP in the waiting room rather than in their own homes was unanticipated, partly because of the reduced convenience. This contrasts with an earlier survey of BP self-monitoring trial participants who preferred to monitor at home rather than in the waiting room.^28^ This variation in findings may result from differences between samples and, in practice, one mode of BP monitoring may not suit everyone.

Unlike BP measurements taken during consultations, self-screening introduces the opportunity for non-disclosure of results by patients to their HCPs. A similar phenomenon has also been observed in patients self-monitoring their BP at home.^32^ Qualitative research found that this was because of perceived lack of interest from the clinician and fear of being prescribed additional medication.^5^ Using a waiting room monitor placed there by the HCPs may diminish the former reason for non-disclosure but HCPs should be aware that their patients may be checking their own BP in the waiting room and not telling them.

### Implications for research and practice

The present results suggest that general practices with BP monitors located in the waiting room may wish to consider the methods by which they promote their presence to patient groups who rarely attend primary care and/or infrequently have their BP measured. They should also reflect on their physical location within the practice to maximise acceptability to patients. Healthcare professionals should also be aware that patients may not report self-screened results unless they are directly asked or there is a clearly signposted system in place to encourage them to submit them for review.

Central to consolidating the role of waiting room monitors in primary care BP screening and hypertension management is confirming their utility. Monitors such as those in most waiting rooms have been clinically validated but no data currently exist to show that such equipment can lead to better rates of diagnosis or control.^6^^–^8 Waiting room monitors could then offer a halfway house for patients unwilling or unable to monitor at home and play a role in diagnostic and management pathways. Future research could evaluate interventions that seek to promote the use of BP self-screening monitors in clinics.

Given the finding that patients screen themselves to confirm their normotensive status, it would be helpful to investigate the impact of elevated self-screened results without an HCP on hand, perhaps using a ‘think aloud’ technique,^33^ in particular to better understand if and when patients decide to disclose such a result. This information would help to better characterise the utility of self-screening as a tool to reduce the burden of untreated hypertension, and the potential for community-based self-screening programmes.

The process of self-screening in the waiting room was generally well liked by service users. It is not currently accessed by all eligible patients, however, because of a lack of awareness about its existence or the perceived stigma of being seen (attempting) to measure BP. Several questions must be answered about BP self-screening in the relatively controlled environment of the waiting room before community BP self-screening should be considered, especially in the face of the limited public demand reported in this study.
